# A systematic review of the magnitude and cause of geographic variation in unplanned hospital admission rates and length of stay for ambulatory care sensitive conditions

**DOI:** 10.1186/s12913-015-0964-3

**Published:** 2015-08-13

**Authors:** John Busby, Sarah Purdy, William Hollingworth

**Affiliations:** 1 School of Social and Community Medicine, University of Bristol, Room 2.07, Canynge Hall, 39 Whatley Road, Bristol, BS8 2PS UK; 2 Professor of Primary Care, School of Social and Community Medicine, University of Bristol, Bristol, UK; 3 Professor of Health Economics, School of Social and Community Medicine, University of Bristol, Bristol, UK

**Keywords:** Geographical distribution, Ambulatory care, Patient admission/sn [Statistics & Numerical Data], Length of stay/sn [Statistics & Numerical Data], Primary care

## Abstract

**Background:**

Unplanned hospital admissions place a large and increasing strain on healthcare budgets worldwide. Many admissions for ambulatory care sensitive conditions (ACSCs) are thought to be preventable, a belief supported by significant geographic variations in admission rates. We conducted a systematic review of the evidence on the magnitude and correlates of geographic variation in ACSC admission rates and length of stay (LOS).

**Methods:**

We performed a search of Medline and Embase databases for English language cross-sectional and cohort studies on 28th March 2013 reporting geographic variation in admission rates or LOS for patients receiving unplanned care across at least 10 geographical units for one of 35 previously defined ACSCs. Forward and backward citation searches were undertaken on all included studies. We provide a narrative synthesis of study findings. Study quality was assessed using a modified Newcastle-Ottawa scale.

**Results:**

We included 39 studies comprising 25 on admission rates and 14 on LOS. Studies generally compared admission rates between regions (e.g. states) and LOS between hospitals. Most of the published research was undertaken in the US, UK or Canada and often focussed on patients with pneumonia, COPD or heart failure. 35 (90 %) studies concluded that geographic variation was present. Primary care quality and secondary care access were frequently suggested as drivers of admission rate variation whilst secondary care quality and adherence to clinical guidelines were often listed as contributors to LOS variation. Several different methods were used to quantify variation, some studies listed raw data, failed to control for confounders and used naive statistical methods which limited their utility.

**Conclusions:**

The substantial geographical variations in the admission rates and LOS of potentially avoidable conditions could be a symptom of variable quality of care and should be a concern for clinicians and policymakers. Policymakers targeting a reduction in unplanned admissions could introduce initiatives to improve primary care access and quality or develop alternatives to admission. Those attempting to curb unnecessarily long LOS could introduce care pathways or guidelines. Methodological work on the quantification and reporting of geographic variation is needed to aid inter-study comparisons.

**Electronic supplementary material:**

The online version of this article (doi:10.1186/s12913-015-0964-3) contains supplementary material, which is available to authorized users.

## Background

Unplanned admissions place a tremendous strain on healthcare resources worldwide. They account for 67 % of hospital bed days at a cost of £12.5bn per year and have risen by 47 % over the last 15 years in England [1] and 13 % between 2000 and 2009 in the USA [2]. Reasons for increases are manifold however demographic changes, increasing pressure on emergency department services, policy changes (e.g. emergency department waiting time targets) and evolving medical practices (e.g. increasing use of day-case surgery) are contributing factors [1]. Concerns that up to 29 % of English unplanned admissions are avoidable [3] have been fuelled by the increasing proportion of short stay admissions, which could be indicative that the admission was unnecessary, and the large variations in admission rates and other process measures between healthcare units [1, 4]. Within a context of shrinking real-terms budgets, healthcare systems worldwide have to deliver care to ageing populations with complex healthcare needs, therefore better understanding of the causes of avoidable admissions is urgently needed.

Opportunities for improved efficiency extend past the initial decision to admit; the length of time a patient spends in hospital, which will ultimately depend on the appropriateness of measures taken to stabilise, diagnose and treat the patient, has substantial clinical and resource implications. Timely discharge is dependent on a range of factors, for example limiting the number of hospital acquired infections [5], but is also heavily reliant on close integration between the hospital and other parts of the health and social care system [6–8]. In the UK, 830,000 bed days were lost in 2013 due to delayed discharge despite patients being medically fit to leave hospital, a figure which represents a rise of 9 % on the previous year [1].

Recent research has focused on reducing admissions due to ambulatory care sensitive conditions (ACSCs) as a means to improve efficiency [9–11]. ACSCs are a subset of diseases where hospital admission is potentially avoidable by preventing the onset of disease (e.g. influenza vaccination), controlling an acute episodic illness (e.g. dehydration and gastroenteritis), or managing a chronic condition effectively (e.g. complications of diabetes) [12].

Observational evidence on the geographic variation in ACSC care could indicate clinical topics where interventions to improve care pathways are most needed and identify regions of the country where quality of care may be poor and further investigation is required. Interpretation of geographic variation is not straightforward and varies by the type of care being studied. Previous work has identified three distinct care types [13]: 1) Effective care where there is strong evidence of efficacy, all well-informed patients would want treatment (e.g. thrombolysis after ischaemic stroke). Geographic variations are likely to reflect underuse in low rate regions. 2) Preference sensitive care where a trade-off between treatments with different risks and benefits is required (e.g. lumpectomy or mastectomy breast cancer treatment). In this situation the appropriate rate could be driven entirely by patient preferences however this is rarely the case, as physician preferences or local practice patterns often play an important role, meaning the optimal rate is unknown and geographic variations are difficult to interpret. 3) Supply sensitive care where there are no consensus on the optimal rate of treatment and rates are largely driven by capacity at the local level. Hospital care for ACSCs is likely to be, at least in part, ‘supply sensitive’ as there is often little evidence on the optimal threshold for admission or appropriate LOS.

The aim of this study is to review the published literature of cross-sectional and cohort studies exploring the magnitude and causes of geographic variation within countries in ACSC admissions and LOS.

## Methods

### Search strategy

We searched all studies included within the Medline (published since 1950) and Embase (published since 1980) databases on 28th March 2013. We used MESH and textword terms for unplanned hospital admission or LOS, geographic variations and observational study designs to identify studies of potential interest. Forward and backward citation searches were undertaken on all studies included from the electronic search using the Web of Science. Full details of the electronic search strategy are given in Additional file 1: Appendix 1.

### Eligibility criteria

Studies were eligible if they used cross-sectional or cohort study designs, were published in English and reported geographic variation in unplanned hospital admission rates or LOS for patients with an ACSC. The list of included ACSCs was based on a systematic review [14] and is provided in Additional file 1: Appendix 2. We included studies which provided any summary measure of geographic variation (e.g. range, coefficient of variation) or studies that provided raw data (e.g. regional rates displayed in tables or plotted on graphs) without any quantitative summary measure. We excluded studies with less than ten geographical units as they were too small to estimate robust measures of variation or identify the drivers of admission rates or LOS. Studies describing admission to the intensive care or emergency department attendance, reporting crude admission counts without accounting for differences in the size of region populations and those set outside OECD countries were also excluded from the analysis. All titles and abstracts were screened for inclusion by one reviewer (JB) and if deemed potentially relevant, full text articles were retrieved. A sample of titles and abstracts, including all those initially selected for full text review and a random sample of those initially excluded, were independently checked by a second reviewer (CC) to assess the reliability of screening.

### Data extraction and quality assessment

One reviewer (JB) extracted data using a standard form. Twenty percent of these were checked by a second reviewer (WH or SP). Data were extracted on the number of admissions, characteristics of patients, number and type of geographical units, statistical methods, results and authors conclusions on the magnitude and causes of variation. Study quality was assessed by the same reviewer (JB) using a modified Newcastle Ottawa Scale for cohort [15] and cross-sectional studies [16].

### Data analysis

We compared the title and abstract screening inter-rater reliability using the kappa statistic [17] and percentage agreement and described the characteristics of the studies included in the review. We analysed studies evaluating admission rates separately from those reporting LOS. The study results were not pooled due to heterogeneous patient populations and statistical methods used to summarise variation. Therefore we carried out a narrative synthesis of the study results. We characterised geographic units as organisational if patients were grouped according to membership or admission to an organisation (e.g. hospital, GP practice) and geographical if patients were grouped into geographical boundaries (e.g. states, primary care trusts). We reported the authors conclusions on the magnitude of variation and separated these into 4 groups (significant variation, variation exists, insignificant variation and no conclusions) based on the strength of the language used. We extracted the causes of variation proposed by the authors, for those tested empirically we extracted the variables tested, for those were causes were hypothesised in the manuscript we extracted the key phrases related to the cause.

## Results

### Search

Of the 5,217 non-duplicate studies retrieved through the electronic search 59 were included after title and abstract screening. A sample of 300 (5.7 %) studies of the 5,217 found during the initial electronic search were checked by a second reviewer; percentage agreement was 97 % and kappa value 0.908 indicating excellent agreement [18].

**Fig 1:**
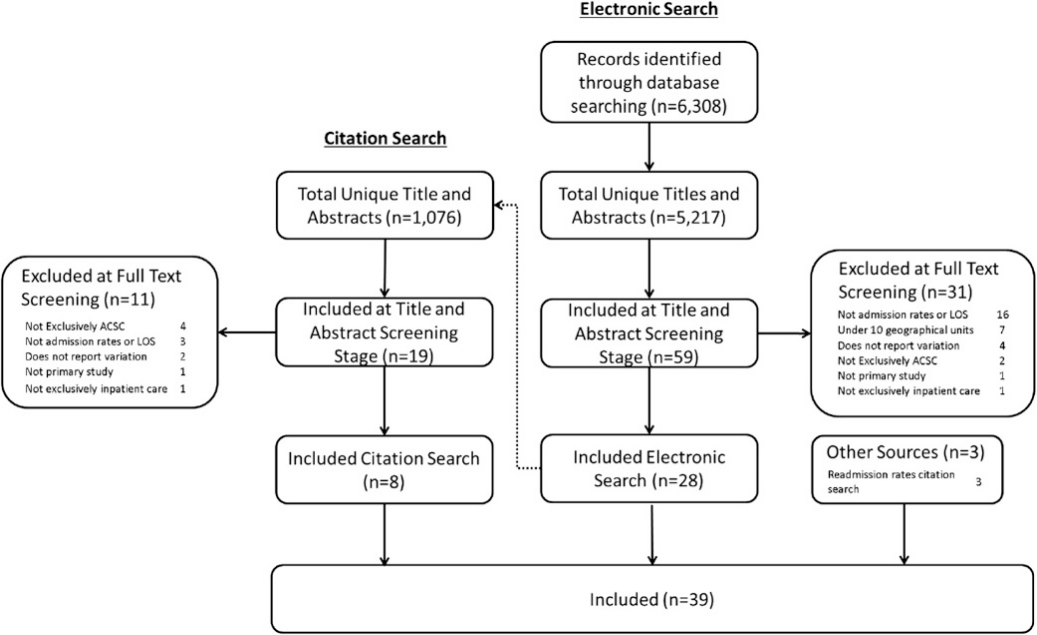
Study flow diagram

Thirty-one studies were excluded during full text screening leaving 28 studies from the initial electronic search (Fig. 1). The most common causes for exclusion were a failure to report data on admission rates or LOS (n = 16; 51.6 %), the study contained less than 10 geographical units (n = 7; 22.6 %) or the study did not report geographic variation (n = 4; 12.9 %). Citation searches of the included studies yielded a further 1,076 titles and abstracts for screening, 8 of which were included. A further 3 eligible studies were found during a separate unpublished systematic review on readmission rates. In total, the review comprised 39 primary studies. There were no substantive differences between reviewers on the 20 % sample of papers there were double extracted, hence the remaining 80 % of studies were extracted by a single reviewer (JB).

### Study quality

A summary of the study quality assessment for the cross-sectional studies investigating geographic variation in admission rates is given in Additional file 1: Appendix 3 and Appendix 4. Studies scored well on the group selection domain as they generally included large sample sizes that were highly representative of patients in the wider community. Scores on the comparability domain were mixed due to a failure to adjust for potentially important case-mix differences (i.e. age and sex) between units. All studies assessed outcomes using medical records which were considered to be at low risk of bias and, as follow-up was only required until discharge for LOS studies, they were generally deemed to be adequate in the outcome domain although the use of naïve statistical methods, for example reporting results using the range, lowered this score in some cases.

### Study characteristics

Twenty-five (64 %) of the included studies reported data on admission rates (Table 1). Five studies (20 %) did not report how many admissions were included, of those that did most were large; 15 of 20 (75 %) were comprised of more than 10,000 patients. Studies varied in both the number (range: 10–3,187) and type of geographic units studied; in 25 of 27 comparisons (93 %) were between geographical units (e.g. counties, states). Studies were set in 5 different countries, with the USA (n = 10; 40 %) commonest, and included several different ACSCs; pneumonia (n = 6; 24 %) was most frequently analysed.

**Table 1:** Study details, admission rates

Paper ID	Condition	Number of Admissions	Geographical Units (N) ^a^	Mean Age (SD)^b^	Covariate Adjustment^c^	Statistical Methods	Tested Cause	Untested Cause
Australia								
Ansari 2005 [56]	Diabetes	38,900	Primary Care Partnerhips (32)	N/A	A,S	Raw Data	None	Case Mix
						Graphs		SC Access
								SC Quality
								Clinical Guidelines
								Coding Quality
Tennant 2000 [57]	Dental	3,754	Health service region (32)	<18 (100 %)	A	Raw Data	None	None
						Graphs		
Canada								
Crighton 2007 [58]	Influenza	241,803	County (49)	N/A	A,S	Raw Data	None	Case Mix
	Pneumonia					Maps		SC Access
						Spatial Analysis		PC Quality
						Range Analysis		Clinical Guidelines
						COV		
Crighton 2008 [59]	Influenza	241,803	County (49)	N/A	A,S	Raw Data	None	Case Mix
						Maps		
								Coding Quality
	Pneumonia							
						Spatial Analysis		
Curtis 2002 [60]	Diabetes	15,872	District Health Board (16)	<18 (100 %)	A,S	Raw Data	None	Case Mix
						Maps		
								PC Quality
						Extremal Quotient		
Jin 2003 [25]	Pneumonia	36,516	Health Region (17)	18-44 (18 %)	A,S	Raw Data	SC Access	Case Mix
				46-64 (19 %)		Graphs		
				65-74 (20 %)				
				75-84 (26 %)				
				85+ (16 %)				
To 1996 [24]	Gastroenteritis	10,105	County (41)	<1 (25.3 %)	A,S	Range	Case Mix	Coding Quality
				1 (25.7 %)		COV	SC Access	
				2 (14.5 %)		SCV		
				3-5 (17.3 %)		Extremal Quotient		
				6-8 (7.0 %)				
				9-14 (4.4 %)				
				12-14 (2.9 %)				
				15-17 (2.8 %)				
New Zealand								
Bandaranayake 2011 [61]	Influenza	1,743	District Health Board (20)	N/A	None	Raw Data	None	Case Mix
						Graphs		
Barnett 2010 [26]	ACSCs	24,894	GP Practice (102)	N/A	None	Raw Data	Case Mix	None
						Graphs	PC Quality	
							PC Access	
							Practice Size	
Dharmalingam 2004 [62]	ACSCs	N/A	Modified District Health Board (29)	N/A	A	Raw Data	Case Mix	None
						Tables		
Ellison-Loschmann 2004 [53]	Asthma	25,865	Territorial Authority (74)	N/A	None	Raw Data	None	Case Mix
						Maps		
Spain								
Magan 2008 [63]	ACSCs	64,409	Health District (34)	78.9	A,S	Raw Data	Case Mix	PC Quality
	Cardiovascular Disease					Maps		Clinical Guidelines
	Heart Failure					Tables		Staffing Levels
	Pneumonia					Range		
						COV		
						SCV		
UK								
Downing 2007 [20]	Asthma	2,271	GP Practice (94)	<65 (84.8 %)	A,S,O	Hierarchical Model	Case Mix	None
	Cardiovascular Disease			>65 (15.2 %)		Variance Estimates	PC Quality	
	COPD							
	Diabetes							
	Stroke							
Giuffrida 1999 [22]	Asthma	N/A	Health Authority (90)	N/A	None	Range	Case Mix	Clinical Guidelines
	Diabetes						SC Access	
							Staffing Levels	
Starr 1996 [56]	Stroke	N/A	Local government districts (22)	40-59 (100 %)	None	Raw Data	Case Mix	SC Access
						Tables		
US								
Adams 1993 [64]	Alcohol Abuse	87,147	State (50)	>65 (100 %)	A,S,O	Raw Data	Case Mix	Coding Quality
						Maps		
Casper 2010 [65]	Heart Failure	N/A	County (3,187)	>65 (100 %)	A	Raw Data	None	Coding Quality
						Maps		PC Access
Chen 2011 [19]	Heart Failure	55,097,390	State (52)	79.0 (7.7)	A,S,C,O	Raw Data	None	None
						Maps		
Gorton 2006 [66]	Pneumonia	4,948	County (67)	59.6 mo	A,S,O	Raw Data	None	Case Mix
						Maps		SC Access
Holt 2011 [67]	COPD	3,786,908	State (50)	>65 (100 %)	None	Raw Data	None	Case Mix
			Hospital Referral Region (949)			Maps		
						Spatial Analysis		
Laditka 1999 [68]	ACSCs	21,923	Hospital Market Area (24)	>65 (100 %)	A,S	Raw Data	None	Case Mix
						Tables		PC Access
Lanska 1994 [69]	Stroke	318,000	State (49)	>65 (100 %)	A,S,O	Raw Data	None	Case Mix
						Maps		SC Access
						Spatial Analysis		SC Access
								Clinical Guidelines
								Procedure/Drug
								Availability
								High readmission rates
Maliszewski 2011 [60]	Influenza	2,010	County (58)	<18 (24.4 %)	A,S,O	Raw Data	Case Mix	None
				>65 (12.4 %)		Maps		
						Spatial Analysis		
Morris 1994 [23]	Asthma	N/A	County (3,079)	>65 (100 %)	A,S,O	Raw Data	Case Mix	Coding Quality
	COPD					Maps	SC Access	
	Pneumonia					Spatial Analysis	Staffing Levels	
Ogunniyi 2012 [70]	Heart Failure	845,421	County (1,014)	65-75 (30.8 %)	A	Raw Data	None	Case Mix
			State (10)	75-84 (41.3 %)		Tables		SC Access
				>85 (27.9 %)		Maps		PC Access
						Spatial Analysis		

^a^Number of geographical units

^b^Mean age and standard deviation when available. Other counts represent percentage of patients in each age band

^c^A: Age, S: Sex, C: Case-Mix, O: Other

^d^Primary Care

^e^Secondary Care

Fourteen studies (36 %) reported data on LOS (Table 2) and were typically smaller than those for admission rates; seven (50 %) included less than 5,000 patients. Studies varied in the number of units compared (range: 10–3,135); the vast majority (n = 13; 93 %) investigated differences between hospitals. Studies were set in six different countries, most commonly the UK (n = 4; 29 %), USA (n = 3; 29 %) and Spain (n = 3; 21 %), while patients with pneumonia (n = 6; 3 %) or COPD (n = 4; 29 %) were most frequently analysed.

**Table 2:** Study details, length of stay

Paper ID	Condition	Number of Admissions	Geographical Units (N)^a^	Mean Age (SD)^b^	Covariate Adjustment^c^	Statistical Methods	Tested Cause	Untested Cause
Belgium								
Claeys 2013 [55]	MI	2,079	Hospital (33)	62 (13)	None	Raw Data	Case Mix	Discharge Planning
						Graphs		
Canada								
Feagan 2000 [71]	Pneumonia	858	Hospital (20)	69.4 (17.7)	A,S,C,O	Raw Data	Case Mix	Clinical Guidelines
						Tables	Hospital Type	PC Access
						% Variation Explained		Procedure/Drug Availability
Denmark								
Klausen 2012 [21]	Pneumonia	12,753	Hospital (22)	65-74 (32.5 %)	A,S,C	Raw Data	Case Mix	Clinical Guidelines
				75-84 (40.6 %)		Graphs	Hospital Size	PC Quality
				>85 (26.9 %)		P-Values (Cox Regression)	Condition Volume	
Spain								
Cabre 2004 [72]	Pneumonia	1,769	Hospital (27)	66.4 (18.1)	A,S,C	Hierarchical Model Variance Estimates	Case Mix	SC Access
								SC Quality
								Clinical Guidelines
								PC Quality
Garau 2008 [73]	Pneumonia	3,233	Hospital (10)	66 (18.5)	A,SC,O	Raw Data	Case Mix	None
						Tables		
						P-Values (Cox Regression)		
Pozo-Rodriguez 2012 [74]	COPD	5,178	Hospital (129)	75 (IQR: 68–80)	None	IQR	None	None
UK								
Hosker 2007 [75]	COPD	8,013	Hospital (233)	71 (IQR: 71–74)	None	IQR	None	None
Price 2006 [27]	COPD	910	Hospital (234)	N/A	A,S,C	IQR	SC Quality	None
						ICC	Clinical Guidelines	
							Hospital Size	
Roberts 2002 [76]	COPD	1,400	Hospital (38)	72	None	Range	Case Mix	SC Quality
						IQR		
Rudd 2001 [77]	Stroke	6,894	Health Region (10)	75 (12)	A,O	Raw Data	Case Mix	None
						Tables	SC Quality	
						P-Values (Kruskal-Wallis)		
US								
Brogan 2012 [78]	Pneumonia	43,819	Hospital (29)	3 (IQR: 1–6)	None	Raw Data	Procedure/Drug Availability	None
						Graphs		
						Range		
Conway 2009 [28]	UTI	20,892	Hospital (25)	1-2 mo (16.7 %)	None	Raw Data	Case Mix	Coding Quality
				2-6 mo (29.9 %)		Graphs	Clinical Guidelines	
				6-24 mo			Condition Volume	
				(19.1 %)				
				2-12 y (34.3 %)				
Drye 2012 [79]	Heart Failure	718,508	Hospital (3,135)	>65 (100 %)	None	Raw Data	None	None
						Graphs		
	MI							
						Range		
	Pneumonia							
Krumholz 1999 [80]	Heart Failure	905	Hospital (49)	<65 (42 %)	A,S,C,O	Raw Data	Case Mix	SC Quality
				>65 (58 %)		Graphs		
						% Variation Explained		

^a^Number of geographical units

^b^Mean age and standard deviation when available. Other counts represent percentage of patients in each age band

^c^A: Age, S: Sex, C: Case-Mix, O: Other

^d^Primary Care

^e^Secondary Care

### Reporting methods and covariate adjustment

Twenty-two (88 %) of the admission rate studies reported raw data using maps (n = 14; 64 %), graphs (n = 5; 23 %) or tables (n = 5; 23 %). Twelve studies (48 %) estimated a summary measure for variation. Most frequently this was with spatial analysis (n = 7; 58 %), such as Morans I^2^, while two studies each reported the coefficient of variation and the systematic component of variation (17 %). Most studies (n = 19; 76 %) adjusted their analysis for covariates that might affect the clinical need for hospital admission, 15 (60 %) studies adjusted for the age and sex of the population, seven of these (28 %) additionally adjusted for case-mix differences or other factors (e.g. deprivation, ethnicity).

Most (n = 9; 64 %) LOS studies reported raw data using graphs (n = 6; 67 %) or tables (n = 3; 33 %). Twelve studies (86 %) estimated a summary measure for variation. Of these the majority were crude measures (e.g. IQR). Only 2 used methods (e.g. hierarchical model variance) that distinguish systematic from random variation. In contrast to admission rate analyses, adjustment for covariates was undertaken in only half of studies (n = 7), five (36 %) adjusted for age, sex and case-mix while one other (7 %) additionally controlled for other factors (i.e. alcohol consumption, blood culture results).

### Magnitude of variation

Two (8 %) studies did not offer conclusions on the magnitude of admission rate variation. Of the remainder 21 (91 %) concluded that some geographic variation was present of which 16 (64 %) reported significant variation (Table 3). For example, one high-quality US study including 55 million heart failure admissions concluded that “Risk-standardized HF hospitalization varied significantly by state” with admission rates ranging from 1,149 to 2,931 per 100,000 person-years [19]. One of the two exceptions which reported no significant variation was based in the UK and found minimal differences in unplanned admission rates for several ACSCs between 94 GP practices after adjustment for age, sex and deprivation differences [20].

**Table 3:** Authors conclusions on variation, admission rates

Paper ID	Author Conclusions
Significant Variation	
Australia	
Ansari 2005	“There was a wide variation (almost fivefold) in admission rates”
Tennant 2000	“[8 of 32 regions] had significantly less episodes of hospitalization…than the State average”
Canada	
Crighton 2007	“Marked differences in rates between counties…large variability in county rates”
Crighton 2008	“The heterogeneity in…hospitalization rates and significant spatial clustering”
New Zealand	
Barnett 2010	“Substantial variation in admission rates”
Dharmalingam 2004	“Substantial geographical variation in the level of avoidable hospitalisation”
Spain	
Magan 2008	“Considerable variability in these rates”
UK	
Giuffrida 1999	“Clear variation…in crude admission rates”
Starr 1996	“There was considerable variation…between districts”
US	
Adams 1993	“There was considerable geographic variation”
Casper 2010	“Magnitude of geographic disparity was substantial between the high- and low-rate counties”
Chen 2011	“Rates in 1998 and 2008 varied significantly by state”
Gorton 2006	“Rates vary widely”
Holt 2011	“Substantial geographic variations in COPD hospitalization risk among states and HSAs”
Laditka 1999	“Significant variation in preventable hospitalization”
Morris 1994	“The geographic distribution in hospital admission rates is unequivocally heterogeneous”
Variation Exists	
Canada	
Curtis 2002	“Differences observed for DKA are clinically important”
Jin 2003	“The incidence of…hospitalization varies”
To 1996	“Variation among the counties…was moderately large”
New Zealand	
Bandaranayake 2011	“We observed a heterogeneous distribution”
US	
Maliszewski 2011	“Hospitalization rates were dependent upon neighbouring county hospitalization rates”
Insignificant Variation	
UK	
Downing 2007	“Generally the variances were small meaning there was little unexplained variation”
US	
Lanska 1994	“Hospitalization rates show relatively little small-scale variation”
No Conclusion	
New Zealand	
Ellison-Loschmann 2004	No conclusions
US	
Ogunniyi 2012	No conclusions

All 13 studies which commented on geographic variation in LOS concluded it existed. Ten (77 %) reported significant variation (Table 4), for example, a high quality Danish study of 11,332 pneumonia admissions at 16 hospitals concluded significant regional differences in median LOS, ranging from two to seven days, were present [21].

**Table 4:** Authors conclusions on variation, length of stay

Paper ID	Author Conclusions
Significant Variation	
Belgium	
Claeys 2013	“Large inter-hospital variations”
Canada	
Feagan 2000	“Considerable heterogeneity in LOSwas noted among the hospitals”
Denmark	
Klausen 2012	“We show significant regional differences”
Spain	
Cabre 2004	“Significant variations…among the 27 community hospitals”
Garau 2008	“Length of stay varied markedly among centres”
UK	
Hosker 2007	“Wide variability between hospitals”
Price 2006	“The wide variation between hospital units…is probably unacceptable”
Roberts 2002	“The variation between hospitals…was very wide”
US	
Conway 2009	“We found high variability in outcomes”
Krumholz 1999	“Significant inter hospital differences in the unadjusted length of stay”
Variation Exists	
UK	
Rudd 2001	“[Length of stay] varied by a mean of eight days between region”
US	
Brogan 2012	“LOS differed across hospitals”
Drye 2012	“Mean patient LOS at the hospital level varied for each condition”
No Conclusion	
Spain	
Pozo-Rodriguez 2012	No conclusions

### Causes of variation

Several reasons for the substantial admission rate variation were proposed (Table 1). Most studies (n = 22; 88 %) noted that unmeasured case-mix differences could be present despite, in many cases, their best efforts at adjustment. Ease of secondary care access (n = 11; 44 %) and inadequate primary care quality (n = 5; 20 %) or access (n = 4; 16 %) were often cited as key drivers of admission rate variation while several studies (n = 6; 24 %) noted that coding quality differences between units could have led to some spurious variation.

Some studies went further and empirically tested the effect of potentially important factors on unplanned admission rates. Increased secondary care access, and in particular bed availability, was reported to be associated with higher admission rates in three studies spanning a wide range of conditions (COPD, asthma, gastroenteritis and diabetes) [22–24] although another study on pneumonia admissions reported no association [25]. Two studies investigated the effect of hospital staffing levels on admission rates with conflicting results. The effect of primary care quality, measured using GP quality scores [20] and the number of GPs within the population [26] respectively, on admission rates was investigated in two studies with no consistent effect. One study found consistent negative associations between staffing levels and admission rates for three respiratory ACSCs [23], which they attribute to improved outpatient care, while another found positive associations for asthma, diabetes and epilepsy admissions [22].

A variety of possible drivers of LOS variation were suggested by authors (Table 2). In accordance with the admission rate results, nine (64 %) of the papers noted that unmeasured case mix differences between units could contribute to the observed variation. The use of clinical guidelines (n = 5; 36 %) and the quality of care received within hospital (n = 5; 36 %) were regularly cited as potentially important factors influencing LOS variation. Some studies went further and empirically investigated the factors that might be important drivers of LOS. A study on UK COPD patients found that higher quality and better organised hospitals, those with more respiratory consultants and those with an early discharge scheme or local written guidelines for follow-up had reduced odds of a length of stay >7 days [27]. A US study of patients with UTI [28] also reported that the presence of clinical practice guidelines is associated with shorter LOS. Three studies investigated the effect of higher condition-specific admissions or hospital size on LOS and found no or very marginal effects [28, 21, 27]. Further details on the causes of variation proposed by authors are given in Additional file 1: Appendix 5 and Appendix 6.

## Discussion

### Main findings

Substantial geographic variation in unplanned ACSCs admission rates and LOS is commonplace in high income countries. A broad literature exists on the topic however, to date, most research has concentrated on pneumonia, heart failure and COPD and been based in the US, UK or Canada. Primary care quality and secondary care access were often cited as drivers of admission rate variation whilst secondary care quality and the use of clinical guidelines were often listed as contributors to LOS variation. Few studies went further and empirically examined the correlates of unplanned admission rates and LOS, results from those that did suggest that increased bed availability and the absence of clinical guidelines may be an important determinant of high admission rates and LOS respectively. Several different methods were used to quantify and report variation, some studies failed to control for confounders and used naive statistical methods which limited their utility.

### Strengths and weaknesses

To our knowledge, this is the first study to systematically review the literature on geographical variation in unplanned ACSC admissions and LOS. Due to resource constraints only a sample of 300 titles and abstract decisions were checked by a second reviewer and 80 % of the full text data extraction was undertaken by a single reviewer which could reduce accuracy. However the high inter-reviewer agreement on title and abstract screening and full-text extraction suggests that our results would not have changed markedly if double screening and extraction of all studies had been undertaken. Our electronic search strategy included only Medline and Embase databases which may have missed some important studies; however our comprehensive Web of Science citation search, which included both forward and backward citations, should limit the effect of this weakness. As many of the terms used to describe geographic variation are non-specific it was difficult to identify relevant articles which may have impaired both the sensitivity and specificity of the electronic search. We have provided a guide on the magnitude of variation present in each study by categorising the authors’ conclusions as ‘significant variation’, ‘variation exists’ and ‘insignificant variation’ however these classifications are subjective.

### Comparison with other studies

A broad international literature exists examining variations in other facets of health care utilisation. Much of the this has built on work by Wennberg and colleagues who, since their seminal paper in 1984 [29], have developed the Dartmouth Atlas [30] in the US which has demonstrated large geographical differences in health care spending, drug use, surgical procedures and care quality over the last 30 years. Other countries have followed with their own Atlases, including England [31, 32] and Spain [33], demonstrating wide levels of variation across their healthcare systems.

Despite the overwhelming evidence that significant geographic variations exist in ACSC admission rates and LOS, relatively little is known about their cause. What little evidence that does exist is in broad agreement with our findings. Secondary care access has been widely documented [13, 34] as an important factor affecting unplanned admission rates. Evidence investigating the link between primary care quality and unplanned admission rates is mixed; two UK based studies found small and inconsistent effects [35, 36] while another found a strong association between high quality and lower cardiovascular admission rates [37]. A recent study also reported substantially decreased admission rates for ACSC conditions where GPs had financial incentives aimed at improving the quality of care [38]. Several studies have demonstrated that clinical guidelines [39] and pathways are effective modifiers of LOS.[40, 41] The effect of secondary care quality on LOS is less clear cut, while some quality improvement initiatives, such as reducing hospital acquired infections,[42] will undoubtedly lead to shorter LOS in some cases higher quality care may necessitate longer LOS [43] leading some to argue that an ‘inverted U’ association between quality and LOS is likely [44], Although issues at the interface between secondary and community care has been highlighted as an important contributor to LOS variation [6–8], few of the studies in our review acknowledged this as an important issue or attempted to measure any association.

### Implications

The substantial geographical variations displayed among the majority of studies in this review should be of concern for policymakers. The large differences, often after adjustment for a range of potential confounders, could be a marker of variable quality care and represent inefficient use of resources. Solutions are not easy, particularly as the optimal unplanned admission rate or LOS is unclear; although it is tempting to assume that lower admission rates and LOS are desirable, it may be that units with very low admission rates or LOS fail their patients if they are denying care to those who would benefit from inpatient treatment or discharging patients inappropriately early, which might result in higher readmission rates downstream [45, 46].

The key question of what can be done to reduce practice variation in potentially avoidable unplanned admissions and LOS is unlikely be answered using observational data however our results alongside the plethora of interventions tested in the wider literature [11] provide some insight. Self-management programmes have been shown to reduce unplanned admissions for patients with COPD [47] and asthma [48] while greater continuity of care with a family physician has been reported to reduce admissions rates [49]. In secondary care, early review by senior clinicians in emergency departments could be effective [50] while the association between increased local bed supply and increased admission rates [34, 23, 24] hints at supply sensitive care [13]. Closer integration between health and social care has shown promising results [51] and has been highlighted as a key aim of the UK government with the introduction of the Better Care Fund [52]. Other interventions such as telemedicine or case management have shown limited or mixed results [11]. Meanwhile, those aiming to curb unnecessary high LOS could introduce secondary care quality improvement initiatives such as clinical pathways [40, 41] or guidelines [39, 28, 27].

]The causes of variation specified by authors also serve to highlight the inherent difficulties with conducting and interpreting studies comparing process measures across several geographical units. Demographic and case-mix differences between units are common problems which require relatively complex statistical models and rich datasets to properly address. Failure to do this, as was the case with several studies included in this review, could lead to biased results. Frustratingly, several studies investigated the effect of case mix variables and, despite finding large associations, still presented unadjusted geographical comparisons [26, 53, 22, 54, 55, 28].

There were wide inconsistencies in the methods used to quantify and report variation. Most studies used graphs, maps or tables without any summary statistic quantifying the extent of variation which does not allow easy comparison between studies. Where summary measures were reported they were often statistically naïve and subject to extreme sampling variability, for example the range and coefficient of variation.

### Unanswered research questions and future research

For future empirical work on variations in ACSC admissions and length of stay to be most useful for policy makers it should fulfil a number of criteria: 1) Compare a large number of regions/units to explore the factors associated with admissions and LOS; 2) Adjust for demography and case mix (prevalence/severity) and acknowledge the potential for unmeasured covariates to introduce bias; 3) Explore other, potentially modifiable, causes of variation (e.g. primary care quality, discharge planning) which might inform policy; 4) Provide a summary measure of variation (e.g. SCV) which delineates random and systematic components of variation; 5) Link to outcomes (e.g. inpatient or 90 day mortality) to make sure that the focus remains on patient outcomes rather than just the cost of the process (fewer admissions, shorter LOS).

## Conclusion

Geographic variation in unplanned ACSCs admission rates and LOS is commonplace in high income countries. These large regional differences should be of concern to policymakers particularly as admissions for these conditions are increasing and are potentially avoidable. Interventions to improve the quality of care and curb variations in practice that cannot be explained by clinical need are urgently needed. Better empirical work quantifying the extent of unexplained variation in ACSCs admission rates and LOS and exploring factors associated more efficient care and better patient outcomes is needed to help design these interventions.

## Additional file

Additional file 1: Appendix 1. Electronic Search Strategy. **Appendix 2.** List of Included Ambulatory Care Sensitive Conditions. **Appendix 3.** Study Quality, Admission Rates. **Appendix 4.** Study Quality, LOS. **Appendix 5.** Causes of Variation, Admission Rates. **Appendix 6.** Causes of Variation, Length of Stay. (DOCX 28 kb)

## Abbreviations

ACSC: ambulatory care sensitive condition
LOS: length of stay
PC: primary care
SC: secondary care
COPD: chronic obstructive pulmonary disease
IQR: inter-quartile range
ICC: inter-class correlation coefficient
COV: coefficient of variation
SCV: systematic component of variation
