# Supplementary material for: A systematic review of the magnitude and cause of geographic variation in unplanned hospital admission rates and length of stay for ambulatory care sensitive conditions
Source: BMC Health Serv Res. 2015 Aug 13;15:324. doi: 10.1186/s12913-015-0964-3 (PMC4535775; doi:10.1186/s12913-015-0964-3)
Supplement: Additional file 1: Appendix 1. — Electronic Search Strategy. Appendix 2. List of Included Ambulatory Care Sensitive Conditions. Appendix 3. Study Quality, Admission Rates. Appendix 4. Study Quality, LOS. Appendix 5. Causes of Variation, Admission Rates. Appendix 6. Causes of Variation, Length of Stay. (DOCX 28 kb) [file 12913_2015_964_MOESM1_ESM.docx]

**Appendix 1: Electronic Search Strategy**

**Database: Medline 1950 to present**

Search Strategy:

--------------------------------------------------------------------------------

1 Hospitalization/ (66091)

2 Patient Admission/ (17285)

3 Patient Readmission/ (6941)

4 "Length of Stay"/ (53203)

5 (("length of" adj3 stay) and (emergenc$ or unscheduled or unplanned or un-planned or unanticipated or unexpected)).tw. (3024)

6 (emergenc$ adj5 (admission$ or readmission$ or hospitali$ or referrral$ or care)).tw. (19359)

7 ((unscheduled or unplanned or un-planned or unanticipated or unexpected) adj5 (admission$ or readmission$ or hospitali$ or care or referral$)).tw. (1481)

8 or/1-7 (147755)

9 small-area analysis/ (898)

10 "Catchment Area (Health)"/ (6614)

11 Geography/ (29004)

12 exp Geography, Medical/ (777)

13 ((difference$ or variation$ or variabilit$ or disparit$) adj5 geographic$).tw. (9131)

14 Healthcare Disparities/ (5904)

15 ((regional$ or region$) adj3 (disparit$ or difference$ or variation$ or variation$)).tw. (20318)

16 "Hospitals"/ (50545)

17 across hospitals.tw. (398)

18 (hospital$ adj3 variation$).tw. (1064)

19 ((difference$ or variation$ or variabilit$ or disparit$) adj5 (admission$ or readmission$ or hospitali$ or ("length of" adj2 stay))).tw. (4252)

20 (coefficient adj2 variation).tw. (14906)

21 ((variation$ or variabilit$ or differen$) adj5 small area$).tw. (288)

22 (differen$ adj2 hospital$1).tw. (4314)

23 or/9-22 (141958)

24 8 and 23 (7162)

25 Epidemiologic studies/ (5601)

26 exp case control studies/ (591562)

27 exp cohort studies/ (1237327)

28 Case control.tw. (64170)

29 (cohort adj (study or studies)).tw. (66473)

30 Cohort analy$.tw. (2934)

31 (Follow up adj (study or studies)).tw. (33870)

32 (observational adj (study or studies)).tw. (33644)

33 Longitudinal.tw. (115753)

34 Retrospective.tw. (226363)

35 Cross sectional.tw. (132721)

36 Cross-sectional studies/ (153284)

37 or/25-36 (1661495)

38 24 and 37 (2575)

**Database: Embase <1980 to 2013 Week 12>**

Search Strategy:

--------------------------------------------------------------------------------

1 Hospitalization/ (186421)

2 Patient Admission/ (96568)

3 Patient Readmission/ (12240)

4 "Length of Stay"/ (71511)

5 (("length of" adj3 stay) and (emergenc$ or unscheduled or unplanned or un-planned or unanticipated or unexpected)).tw. (4954)

6 (emergenc$ adj5 (admission$ or readmission$ or hospitali$ or referrral$ or care)).tw. (26909)

7 ((unscheduled or unplanned or un-planned or unanticipated or unexpected) adj5 (admission$ or readmission$ or hospitali$ or care or referral$)).tw. (2281)

8 or/1-7 (347857)

9 ((difference$ or variation$ or variabilit$ or disparit$) adj5 geographic$).tw. (11219)

10 ((regional$ or region$) adj3 (disparit$ or difference$ or variation$ or variation$)).tw. (24517)

11 across hospitals.tw. (518)

12 (hospital$ adj3 variation$).tw. (1397)

13 ((difference$ or variation$ or variabilit$ or disparit$) adj5 (admission$ or readmission$ or hospitali$ or ("length of" adj2 stay))).tw. (6406)

14 (coefficient adj2 variation).tw. (17917)

15 ((variation$ or variabilit$ or differen$) adj5 small area$).tw. (342)

16 (differen$ adj2 hospital$1).tw. (6236)

17 geography/ (31535)

18 medical geography/ (30)

19 health care disparity/ (4485)

20 or/9-19 (99695)

21 8 and 20 (7733)

22 Clinical study/ (45529)

23 Case control study/ (74810)

24 Longitudinal study/ (59025)

25 Retrospective study/ (311538)

26 Cohort analysis/ (142457)

27 (Cohort adj (study or studies)).tw. (92900)

28 (Case control adj (study or studies)).tw. (67231)

29 ("follow up" adj (study or studies)).tw. (41561)

30 (observational adj (study or studies)).tw. (51901)

31 (epidemiologic$ adj (study or studies)).tw. (68662)

32 (cross sectional adj (study or studies)).tw. (69952)

33 "cross-sectional study"/ (89818)

34 "major clinical study"/ (1695596)

35 "Comparative Study"/ (695110)

36 or/22-35 (2906048)

37 21 and 36 (4371)

38 limit 37 to embase (3733)

39 limit 38 to conference abstract (302)

40 38 not 39 (3431)

[conference abstracts downloaded separately to other records]

**Appendix 2: List of Included Ambulatory Care Sensitive Conditions**

| **Condition** |  |
| --- | --- |
| Alcohol-related diseases | Hypertension |
| Angina | Hypokalemia |
| Asthma | Influenza and pneumonia |
| Atrial fibrillation and flutter | Iron-deficiency anaemia |
| Cellulitis | Low birth weight |
| Chronic obstructive pulmonary disease | Migraine / acute headache |
| Heart failure | Neuroses |
| Constipation | Nutritional deficiency |
| Convulsions and epilepsy | Other vaccine-preventable diseases |
| Dehydration and gastroenteritis | Pelvic inflammatory disease |
| Deliberate self-harm | Peripheral vascular disease |
| Dental Conditions | Pyelonephritis |
| Diabetes complications | Ruptured appendix |
| Dyspepsia and other stomach function disorders | Schizophrenia |
| Ear, nose and throat infections | Senility / dementia |
| Failure to thrive | Stroke |
| Fractured proximal femur | Tuberculosis |
| Gangrene |  |

**Appendix 3: Study Quality^f^, Admission Rates**

| **Paper ID** | **Selection** | | | |  | **Comparability** |  | **Outcome** | |
| --- | --- | --- | --- | --- | --- | --- | --- | --- | --- |
|  | 1 | 2 | 3 | 4 |  | 1 |  | 1 | 2 |
| **Australia** |  |  |  |  |  |  |  |  |  |
| Ansari 2005 | ✓ | ✓ | ✓ | ✓ |  | ✓ |  | ✓ | ✓ |
| Tennant 2000 | ✓ | ✓ | ✓ | ✓ |  | 🗶 |  | ✓ | ✓ |
| **Canada** |  |  |  |  |  |  |  |  |  |
| Crighton 2007 | ✓ | ✓ | ✓ | ✓ |  | ✓ |  | ✓ | ✓ |
| Crighton 2008 | ✓ | ✓ | ✓ | ✓ |  | ✓ |  | ✓ | ✓ |
| Curtis 2002 | ✓ | ✓ | ✓ | ✓ |  | ✓ |  | ✓ | ✓ |
| Jin 2003 | ✓ | ✓ | ✓ | ✓ |  | ✓ |  | ✓ | ✓ |
| To 1996 | ✓ | ✓ | ✓ | ✓ |  | ✓ |  | ✓ | ✓ |
| **New Zealand** | |  |  |  |  |  |  |  |  |
| Bandaranayake 2011 | ✓ | ✓ | ✓ | ✓ |  | 🗶 |  | ✓ | ✓ |
| Barnett 2010 | ✓ | ✓ | ✓ | ✓ |  | 🗶 |  | ✓ | ✓ |
| Dharmalingam 2004 | ✓ | ✓ | ✓ | ✓ |  | 🗶 |  | ✓ | ✓ |
| Ellison-Loschmann 2004 | ✓ | ✓ | ✓ | ✓ |  | 🗶 |  | ✓ | ✓ |
| **Spain** |  |  |  |  |  |  |  |  |  |
| Magan 2008 | ✓ | ✓ | ✓ | ✓ |  | ✓ |  | ✓ | ✓ |
| **UK** |  |  |  |  |  |  |  |  |  |
| Downing 2007 | ✓ | ✓ | ✓ | ✓ |  | ✓ |  | ✓ | ✓ |
| Giuffrida 1999 | ✓ | ✓ | ✓ | ✓ |  | 🗶 |  | ✓ | 🗶 |
| Starr 1996 | ✓ | ✓ | ✓ | ✓ |  | 🗶 |  | ✓ | ✓ |
| **US** |  |  |  |  |  |  |  |  |  |
| Adams 1993 | ✓ | ✓ | ✓ | ✓ |  | ✓ |  | ✓ | ✓ |
| Casper 2010 | ✓ | ✓ | ✓ | ✓ |  | 🗶 |  | ✓ | ✓ |
| Chen 2011 | ✓ | ✓ | ✓ | ✓ |  | ✓ |  | ✓ | ✓ |
| Gorton 2006 | ✓ | ✓ | ✓ | ✓ |  | ✓ |  | ✓ | ✓ |
| Holt 2011 | ✓ | ✓ | ✓ | ✓ |  | 🗶 |  | ✓ | ✓ |
| Laditka 1999 | ✓ | ✓ | ✓ | ✓ |  | ✓ |  | ✓ | ✓ |
| Lanska 1994 | ✓ | ✓ | ✓ | ✓ |  | ✓ |  | ✓ | ✓ |
| Maliszewski 2011 | ✓ | ✓ | ✓ | ✓ |  | ✓ |  | ✓ | ✓ |
| Morris 1994 | ✓ | ✓ | ✓ | ✓ |  | ✓ |  | ✓ | ✓ |
| Ogunniyi 2012 | ✓ | ✓ | ✓ | ✓ |  | 🗶 |  | ✓ | ✓ |

Assessed using the Newcastle-Ottawa Scale for cross-sectional studies Selection: 1) Representativeness of the sample 2) Sample size 3) Non-respondents 4) Ascertainment of the exposure Comparability: 1) The subjects in different outcome groups are comparable, based on the study design or analysis. Confounding factors are controlled. Outcome: 1) Assessment of the outcome 2) Statistical test

**Appendix 4: Study Quality^g^, Length of Stay**

| **Paper ID** | **Selection** | | | |  | **Comparability** |  | **Outcome** | | | |
| --- | --- | --- | --- | --- | --- | --- | --- | --- | --- | --- | --- |
|  | 1 | 2 | 3 | 4 |  | 1 |  | 1 | 2 | 3 | 4 |
| **Belgium** |  |  |  |  |  |  |  |  |  |  |  |
| Claeys 2013 | ✓ | ✓ | ✓ | ✓ |  | 🗶 |  | ✓ | ✓ | ✓ | ✓ |
| **Canada** |  |  |  |  |  |  |  |  |  |  |  |
| Feagan 2000 | 🗶 | ✓ | ✓ | ✓ |  | ✓ |  | ✓ | ✓ | ✓ | ✓ |
| **Denmark** |  |  |  |  |  |  |  |  |  |  |  |
| Klausen 2012 | ✓ | ✓ | ✓ | ✓ |  | ✓ |  | ✓ | ✓ | ✓ | ✓ |
| **Spain** |  |  |  |  |  |  |  |  |  |  |  |
| Cabre 2004 | ✓ | ✓ | ✓ | ✓ |  | ✓ |  | ✓ | ✓ | ✓ | ✓ |
| Garau 2008 | 🗶 | ✓ | ✓ | ✓ |  | ✓ |  | ✓ | ✓ | ✓ | ✓ |
| Pozo-Rodriguez 2012 | ✓ | ✓ | ✓ | ✓ |  | 🗶 |  | ✓ | ✓ | ✓ | 🗶 |
| **UK** |  |  |  |  |  |  |  |  |  |  |  |
| Hosker 2007 | ✓ | ✓ | ✓ | ✓ |  | 🗶 |  | ✓ | ✓ | ✓ | 🗶 |
| Price 2006 | 🗶 | ✓ | ✓ | ✓ |  | ✓ |  | ✓ | ✓ | ✓ | 🗶 |
| Roberts 2002 | 🗶 | ✓ | ✓ | ✓ |  | 🗶 |  | ✓ | ✓ | ✓ | 🗶 |
| Rudd 2001 | ✓ | ✓ | ✓ | ✓ |  | ✓ |  | ✓ | ✓ | ✓ | ✓ |
| **US** |  |  |  |  |  |  |  |  |  |  |  |
| Brogan 2012 | 🗶 | ✓ | ✓ | ✓ |  | 🗶 |  | ✓ | ✓ | ✓ | ✓ |
| Conway 2009 | 🗶 | ✓ | ✓ | ✓ |  | 🗶 |  | ✓ | ✓ | ✓ | ✓ |
| Drye 2012 | ✓ | ✓ | ✓ | ✓ |  | 🗶 |  | ✓ | ✓ | ✓ | ✓ |
| Krumholz 1999 | 🗶 | ✓ | ✓ | ✓ |  | ✓ |  | ✓ | ✓ | ✓ | ✓ |

Assessed using the Newcastle-Ottawa Scale for cohort studies Selection: 1) Is the case definition adequate, 2) Representativeness of the cases 3) Selection of controls 4) Definitions of controls

Comparability: 1) Comparability of cases and controls on the basis of the design or analysis

Outcome: 1) Ascertainment of outcome 2) Adequate follow-up time 3) Completeness of follow-up 4) Statistical test

**Appendix 5: Further details on causes for variation for admission rate studies**

| **Paper ID** | **Cause** | **Variables / Statement** |
| --- | --- | --- |
| **Australia** |  |  |
| Ansari 2005 | Case Mix | “Possible contributing factors to higher rates of admissions are...propensity of individuals to seek care, cultural factors, the prevalence of diabetes" |
|  | SC Access | “Possible contributing factors to higher rates of admissions are...relative scarcity of outpatient murces" |
|  | SC Quality | “Possible contributing factors to higher rates of admissions are...quality issues" |
|  | Clinical Guidelines | “Possible contributing factors to higher rates of admissions are...hospital admission practices" |
|  | Coding Quality | “Diagnoses recorded in the VAED are subject to coding errors" |
| **Canada** |  |  |
| Crighton 2007 | Case Mix | “Locally, ‘hot spots’ were identified in several northern rural counties and ‘cold spots ’ in southern urban counties" |
|  | SC Access | “Other potential geographically variable factors associated with the decision to hospitalize such as...bed availability" |
|  | Clinical Guidelines | ““[The high degree of variability in pneumonia and influenza rates among the younger age groups] is probably explained by hospitalization criteria" |
|  | PC Quality | “Better access to home care and emergency medical services [may explain part of the urban/rural pattern]" |
| Crighton 2008 | Case Mix | “The aggregated ICD-9 codes used in this analysis consist of a variety of bacterial and viral pneumonias...[which] could also be expected to vary in their geographic distribution" |
|  | Coding Quality | “[This analysis] has revealed data quality issues resulting from limited diagnostic capacity and crude diagnostic codes" |
| Curtis 2002 | Case Mix | “The more populated urban areas had lower rates of both DKA and non-DKA admissions compared with the more remote sparsely populated regions" |
|  | PC Quality | “This large urban area likely provides greater access to both primary and tertiary care than more remote regions" |
| Jin 2003 | SC Access* | Hospital beds per capita |
|  | Case Mix | “The rate of hospital discharges is greater than the provincial average in rural health regions" |
| To 1996 | Case Mix* | % Literacy, % living on reservation, % income below $35,000, % dwelling need major repairs |
|  | SC Access* | Hospital beds per capita |
|  | Coding Quality | “There may also be coding differences between regions that account for differences in admissions" |
| **New Zealand** | |  |
| Bandaranayake 2011 | Case Mix | “Some regions (mainly small urban and rural areas) that had relatively low ILI activity" |
| Barnett 2010 | Case Mix* | % Patients 0-4, % Patients 65 and over, Males per 100 females, Mean deprivation, % Maori, % Asian |
|  | PC Quality* | Care Plus Enrolment |
|  | PC Access* | Patients per GP |
|  | Practice Size* | GP practice list size |
| Dharmalingam 2004 | Case Mix* | % Maori |
| Ellison-Loschmann 2004 | Case Mix | “Overall the relative risk of hospitalisation was higher in urban than in rural TAs" |
| Spain |  |  |
| Magan 2008 | Case Mix* | Female |
|  | Clinical Guidelines | “[High correlation between males and females] may indicate the existence of a common factor such as different admission policies in reference hospitals" |
|  | PC Quality | “[High correlation between males and females] may indicate the existence of a common factor such as deficiencies in PHC" |
|  | Staffing Levels | “It would be useful...to identify the factors such as number of physicians...that may influence variability" |
| **UK** |  |  |
| Downing 2007 | Case Mix* | Female, Age |
|  | PC Quality* | QoF [Quality outcomes framework] additional services score, QoF clinical score, QoF Organisational score |
| Giuffrida 1999 | Case Mix* | Population density, Unemployment, No Central Heating, Crowded accommodation, No car, New Commonwealth, Retired living alone, Students, Social class I and II, Population mobility |
|  | SC Access* | Hospital beds per capita |
|  | Staffing Levels* | General physicians per capita |
|  | Clinical Guidelines | “There are no data available that would enable allowance to be made for...the admission policies of individual hospital" |
| Starr 1996 | Case Mix* | Population density, Urban, Deprivation, % smokers, Systolic blood pressure, Diastolic blood pressure, Cholesterol, HDL, Triglyceride, Fibrinogen, BMI, Exercise at work, Exercise at home, Alcohol consumption, % not eating fruit |
|  | SC Access | “Residents may be more readily admitted to hospital compared with people in rural areas simply because of the proximity of secondary care facilities" |
| **US** |  |  |
| Adams 1993 | Case Mix* | Alcohol consumption |
|  | Coding Quality | “There is variability among hospitals and from state to state in the accuracy of diagnostic coding" |
| Casper 2010 | Coding Quality | “If there are geographic differences in financial incentives to report HF as the first-listed diagnosis, then these results could be explained by that coding bias" |
|  | PC Access | “High rates of HF hospitalizations may identify counties that are in particular need of improved access to quality health care" |
| Gorton 2006 | Case Mix | “Some counties with high admission rates may confront risk factors for which our analysis could not control adequately" |
|  | SC Access | “Some Pennsylvania counties with low rates may face inadequate access" |
| Holt 2011 | Case Mix | “These findings suggest that two spatial processes may be operating with respect to environmental influences on COPD hospitalization; first, broad regionalized contextual effects (e.g. socioeconomic factors and high regionalized population smoking rates) may be exacerbating COPD hospitalizations. Second, localized environmental factors, such as occupational exposures, may be influencing COPD hospitalizations" |
| Laditka 1999 | Case Mix | “All of the intra-county areas having significantly higher than average rates of preventable hospitalization for older women and men were classified as low income areas" |
|  | PC Access | “These findings indicate that when potential access problems for primary care exist for older persons in specific geographic areas" |
| Lanska 1994 | Case Mix | “Hospital usage rates are potentially influenced by…incidence of stroke, frequency of comorbidities" |
|  | SC Access | “Hospital usage rates are potentially influenced by…bed availability" |
|  | SC Access | “Hospital usage rates are potentially influenced by...access to health" |
|  | Clinical Guidelines | “Hospital usage rates are potentially influenced by…hospital admission policies and practices" |
|  | Procedure / Drug Availability | “Hospital usage rates are potentially influenced by…diagnostic and therapeutic fashions and capabilities" |
|  | High readmission rates | “Hospital usage rates are potentially influenced by…high readmission rates" |
| Maliszewski 2011 | Case Mix* | Age, Race, Sex, Socioeconomic status |
| Morris 1994 | Case Mix* | Income, School years, Household crowding, Population density, Lung Cancer Incidence, Occupational lung disease, Average annual temperature |
|  | SC Access* | Hospital beds per capita |
|  | Staffing Levels* | Physicians per capita |
|  | Coding Quality | “An alternative explanation for the observed heterogeneity in the distribution of hospital admission rates is regional differences in coding of Medicare data" |
| Ogunniyi 2012 | Case Mix | “HF hospitalization rates among Medicare beneficiaries in the catchment area were generally higher in rural areas than in urban areas" |
|  | SC Access | “Possible explanations for this difference include generally greater access to physicians in urban areas" |
|  | PC Access | “HF rates were generally lower in counties with high PCP [primary care practitioner] -to-beneficiary ratios" |
| * Empirically tested | |  |

**Appendix 6: Further details on causes for variation for LOS studies**

| **Paper ID** | **Cause** | **Variables / Statement** |
| --- | --- | --- |
| **Belgium** |  |  |
| Claeys 2013 | Case Mix* | Age, Female, Killip class >1, PAD, Anterior infarction, Ischaemic time> 4h, No reperfusion treatment |
|  | Clinical Guidelines | “These [inter-hospital] differences seem to be related to differences in general discharge policies" |
| **Canada** |  |  |
| Feagan 2000 | Case Mix* | PSI Risk Class |
|  | Hospital Type* | Teaching Hospital |
|  | Clinical Guidelines | “Although the incorporation of such interventions [uniform discharge criteria] into practice guidelines might decrease the variation in LOS" |
|  | PC Access | “Current Canadian health care policy could also be contributing to the variation in LOS... the restrictions on the availability of home care services [may increase LOS]" |
|  | Procedure / Drug Availability | “...the incorporation of such interventions [oral antibiotic therapy] into practice guidelines might decrease the variation in LOS" |
| **Denmark** |  |  |
| Klausen 2012 | Case Mix* | Men, Age, Ventilator support, Carlson Index |
|  | Hospital Size* | Small vs. Large |
|  | Condition Volume* | Few vs. Many |
|  | Clinical Guidelines | “Differences between regions in recommendations for discharge could be a potential explanation for the observed regional differences in LOS" |
|  | PC Quality | “Differences between regions in… cooperation with the primary healthcare system could be a potential explanation for the observed regional differences in LOS" |
| **Spain** |  |  |
| Cabre 2004 | Case Mix* | PSI Risk Class, Complications, Admission to ICU, Oxygen therapy, Discharge to nursing home |
|  | SC Access | “[The causes for significant inter-hospital differences include] rate of occupancy of hospital beds" |
|  | SC Quality | “[The causes for significant inter-hospital differences include] physicians’ skills, experience and competence" |
|  | Clinical Guidelines | “[The causes for significant inter-hospital differences include] use of good proven clinical guidelines" |
|  | PC Quality | “[The causes for significant inter-hospital differences include] health system accessibility and primary health-care support" |
| Garau 2008 | Case Mix* | PSI Risk High, Blood Cultures Positive, ICU Admittance, Empirical antibiotic, X-Ray multi-lobar, Aetiological diagnosis, Active tobacco user, Regular alcohol consumption |
| **UK** |  |  |
| Price 2006 | SC Quality* | Hospital star rating |
|  | Clinical Guidelines* | Guideline for follow up, Early discharge scheme |
|  | Hospital Size* | Bed numbers |
|  | Staffing Levels* | No. respiratory consultants |
| Roberts 2002 | Case Mix* | Age, FEV, Performance status, Admission PEF, Arterial CO2, IPPV or NPPV Initial Management |
|  | SC Quality | “The variation between hospitals for each of the process indicators described previously was very wide, suggesting that care standards vary widely" |
| Rudd 2001 | Case Mix* | Age |
|  | SC Quality* | Management on stroke ward |
| **US** |  |  |
| Brogan 2012 | Procedure / Drug Availability* | Number of diagnostic tests |
| Conway 2009 | Case Mix* | Age, Race, Hispanic ethnicity, Insurance, Hospital High % Medicad / uninsured |
|  | Clinical Guidelines* | Presence of guidelines |
|  | Condition Volume* | UTI admission volume |
|  | Coding Quality | “However, the ICD-9 codes may not have captured all patients with co-morbidities" |
| Krumholz 1999 | Case Mix* | Age, Female, White race, Admitted from SNF, Admitted from ED, Cardiologist attending, Prior renal failure, Worsening heart failure, Peripheral edema, Atrial fibrillation, Sodium<135 mmol/L, BUN:creatine>20, Admitted to ICU/CCU, Urinary catheter on admission, New use of antiarrhythmic, New use of digoxin, New use of ace INHIBITOR, New use of warfarin, Major complications |
|  | SC Quality | “Differences in hospital stay may also depend on...the vigilance of the nursing team" |
| * Empirically tested | |  |
